# The role of immune surveillance in malignant transformation of benign salivary gland tumors

**DOI:** 10.18632/oncotarget.27900

**Published:** 2021-03-30

**Authors:** Maximilian Linxweiler, Jingming Wang, Luc G.T. Morris

**Affiliations:** ^1^Department of Otorhinolaryngology, Head and Neck Surgery, Saarland University Medical Center, Homburg/Saar, Germany; ^2^Immunogenomics and Precision Oncology Platform, Memorial Sloan Kettering Cancer Center, New York, NY, USA; ^3^Department of Surgery, Memorial Sloan Kettering Cancer Center, New York, NY, USA

**Keywords:** salivary tumor, salivary cancer, pleomorphic adenoma, immune surveillance

## Abstract

Pleomorphic adenoma (PA), the most common salivary gland tumor, is a benign tumor that carries a risk of malignant transformation to various histologies of carcinoma ex pleomorphic adenoma (CA exPA). Recently, genomic analyses have provided deeper insights into the molecular biology of salivary gland cancers. However, the molecular processes that underlie the progression from PA to CA exPA are largely unknown. In this study, we used RNAseq data from CA ex PA of myoepithelial (*n* = 24) or salivary duct histology (*n* = 6), *de novo* myoepithelial carcinoma (*n* = 16) and *de novo* salivary duct carcinoma (*n* = 10), and compared their constituent immune tumor microenvironments. We found that increasing levels of immune infiltration and activation were associated with a generally lower probability of cancer developing ex-PA, suggesting that immune surveillance may constrain the malignant transformation of benign salivary tumors. More immunologically infiltrated tumors were more likely to have developed *de novo*. Taken together, these data suggest a role for tumor escape from immune surveillance in the development of CA exPA. The immune-cold microenvironments of CA ex PA tumors may in part explain their more aggressive clinical behavior.

## INTRODUCTION

Salivary gland cancers (SGCs) are rare, aggressive human malignancies that account for fewer than 5% of all carcinomas arising within the head and neck. These difficult-to-treat cancers exhibit a wide spectrum of morphological and histopathological characteristics: more than 20 distinct SGC subtypes arise within salivary gland tissue [1]. Therapeutic options are limited: beyond surgery and radiotherapy in curative treatment settings, no standard therapy exists for recurrent and metastatic disease, where chemotherapy regimens have generally demonstrated low rates of response and lack of clear clinical benefit [1, 2].

Over the past several years, genomic analyses have provided deeper insights into the molecular biology of SGCs and led to the identification of potentially druggable genetic alterations including mutations in *PIK3CA* and *BRAF, ETV6-NTRK3* fusions, and overexpression of Her-2 and androgen receptors [3, 4]. However, due to the rarity of SGCs, clinical evidence in support of these therapeutic concepts has thus far been limited to early phase trials or basket studies with small patient cohorts.

Carcinoma ex pleomorphic adenoma (CA exPA) is a rare malignant neoplasm of the major salivary glands, accounting for about 3–15% of all SGCs [2, 5], with rising incidence over the past several decades [5, 6]. CA exPA is the result of malignant transformation of the most common benign salivary gland tumor—pleomorphic adenoma (PA)—into malignant histologies such as adenocarcinoma (AC), salivary duct carcinoma (SDC), adenoid cystic carcinoma (ACC), and myoepithelial carcinoma (MECA) [7]. This transformation generally occurs after the PA has been present for many years, usually decades. The prognosis of salivary malignancies that have evolved from a pleomorphic adenoma is generally worse than that of *de novo* salivary gland malignancies, with a reported 5-year overall survival-rate as low as 25% [1]. Major prognostic factors include tumor size, lymphatic and distant metastasis, grading, proportion of carcinoma, proliferation index, and extent of invasion [1, 5, 7].

Recent molecular analysis of CA exPA have generated new data shedding light on mechanisms of carcinogenesis. These studies have identified prevalent mutations of *TP53* as well as high expression levels of cyclin D1, p16, COX-2, HGF-A, c-Met, and EGFR [1]. However, for the most part, the landscape of mutational and copy number alterations has been observed to be largely similar to non-malignant pleomorphic adenomas [8], leaving the molecular processes involved in the transition from PA to CA exPA still elusive.

Recently we analyzed RNA sequencing (RNAseq) data from SGC tumors to better understand the immune tumor microenvironment (TME) of these cancers. These analyses have shown that the immune TME varies widely across the different histologic subtypes of SGC. We deconvolved and analyzed the immune microenvironment and neoantigen landscape in 76 SGCs representing the three most lethal histologies: MECA (*n* = 40), ACC (*n* = 20), and SDC (*n* = 16) [9].

On one end of the spectrum, SDCs generally exhibit high levels of immune infiltration with concomitant higher levels of T-cell dysfunction, as well as higher mutational load. In stark contrast, ACCs tended to have an immune-excluded microenvironment, with immunosuppressive cellular populations such as M2-polarized macrophages and myeloid-derived suppressor cells both prevalent, in addition to harboring a very low mutational load. MECAs had a more heterogeneous, but generally intermediate, pattern, with both immune-low and immune-high phenotypes. Taken together, these data provided new insights into the immune microenvironment and neoantigen landscape of SGCs, showing that mechanisms of immune escape appear to differ by histology.

To our knowledge, no studies have examined the potential role of the immune microenvironment in modulating the evolution of PA to CA exPA. Here, to evaluate a possible association between immune surveillance and the development of CAexPA, we used RNA sequencing data from the above SGC cohort with a focus on 24 MECA and 6 SDC cases that developed from PA.

## RESULTS

We used bulk RNAseq data for immune deconvolution applying several orthogonal tools including CIBERSORT, ssGSEA and ESTIMATE as described in detail previously [9].

**Figure 1:**
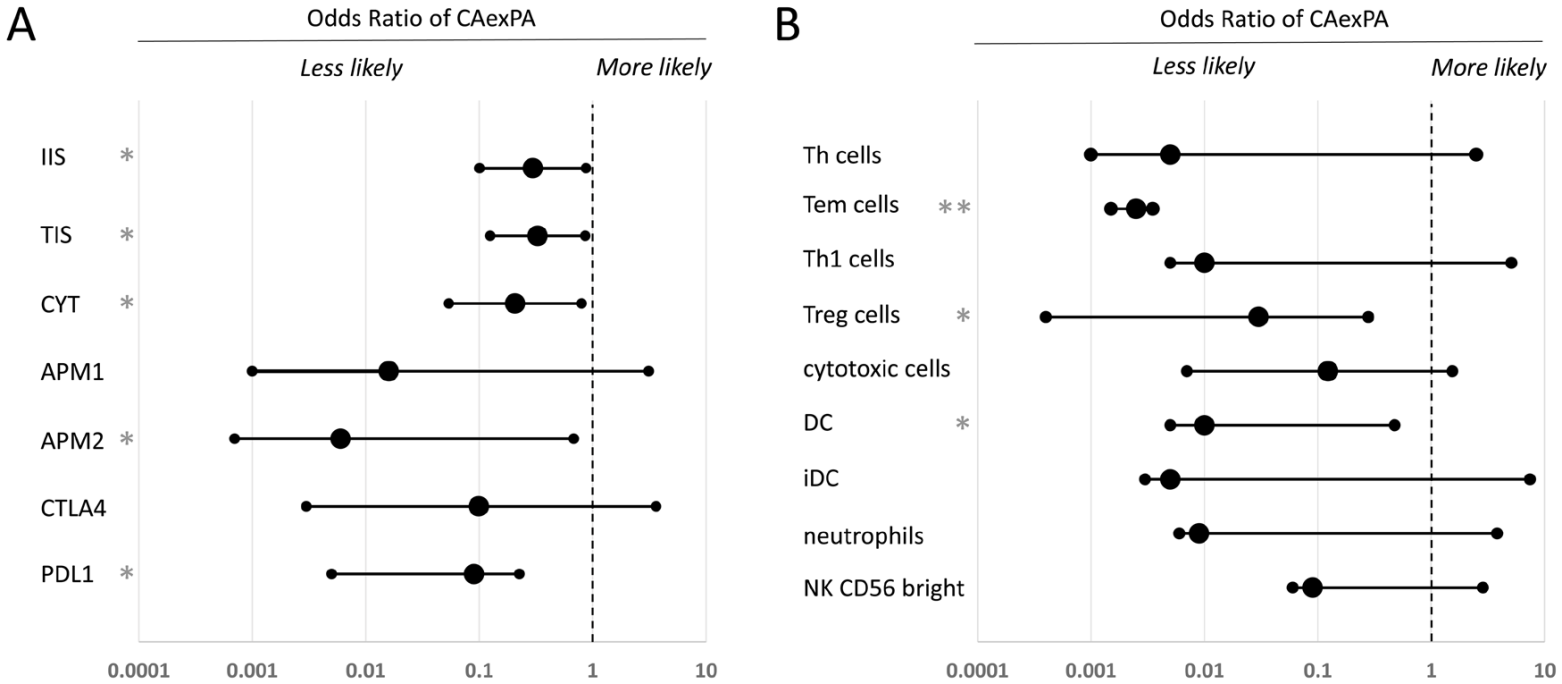
Comparative analysis of the immune microenvironment in carcinoma ex-pleomorphic adenoma versus *de novo* salivary gland carcinoma. Logistic regression was used to analyze associations between the tumor immune microenvironment and ex-pleomorphic adenoma versus *de novo* tumor status, controlling for cancer histology as a covariate. (**A**) Odds ratio of the cancer being exPA for aggregate markers of immune infiltration and activity. (**B**) Odds ratio of the cancer being exPA for specific immune cell populations. In (A) and (B), error bars indicate the 95% confidence interval. Statistically significant results are labeled (^*^ *p* < 0.05; ^**^ *p* < 0.005). PDL1: programmed cell death 1 ligand; CTLA4: Cytotoxic T-lymphocyte-associated protein 4; APM2: MHC class II antigen processing machinery; APM1: MHC class I antigen processing machinery; CYT: cytolytic score; TIS: T cell infiltration score; IIS: immune infiltration score; NK cells: natural killer cells; iDC: immature dendritic cells; DC: dendritic cells; Treg cells: regulatory T cells; Th1 cells: Type 1 T helper cells; Tem cells: T effector memory cells; Th cells: T helper cells; OR: Odds Ratio; C.I: confidence interval.

In a logistic regression model controlling for histology type as covariate, we analyzed the association between immunological tumor microenvironment metrics and cancer origin in MECA (*n* = 24) and SDC ex PA (*n* = 6) and *de novo* MECA (*n* = 16) and SDC (*n* = 10). In general, we observed directionally consistent associations between all aggregate immune metrics and the probability of cancer developing ex-PA (Figure 1A). These data are consistent with more immunologically infiltrated tumors being less likely to develop from a PA, and more likely to develop *de novo*. While these data cannot confirm any specific causal mechanism, they suggest that immune surveillance may constrain the transformation of longstanding benign PA tumors to CA exPA, and that this phenomenon is less relevant in *de novo* carcinogenesis, where immune escape may be required early on in tumor development.

We then performed an exploratory analysis to determine whether any individual immunological cellular populations or processes were potentially associated with this phenotype (Figure 1B). Because these data are hypothesis-generating, we caution that these analyses did not include correction for multiple hypothesis testing. All cellular populations were associated with a lower likelihood of CA exPA, although the associations were only statistically significant for T cells, Tem cells, Treg cells, DCs, and MHC type II antigen processing machinery.

Taken together, these data reveal a more immune-depleted or immune-excluded microenvironment in MECAs and SDCs that developed from PA compared to MECAs and SDCs that developed *de novo*. Because prior studies have indicated that the mutational landscape in PA and CA exPA are largely similar, it is quite possible that immune escape is a necessary step in the malignant transformation of PA tumors, which do not transform so long as they remain under effective adaptive immune surveillance.

These data are consistent with the prior findings reported by Furuse and colleagues, who found high levels of TGFß expression in 10/10 cases of CA exPA; in contrast to absent expression in the epithelial and myoepithelial cells of PA [10]. As TGFß represents a key regulator of immune evasion and immune suppression in human cancer, this observation aligns with our hypothesis that defects in immune surveillance may contribute to the transformation of PA into CA exPA. However, it is important to qualify that high TGFß expression may be a consequence rather than a cause of CA exPA carcinogenesis.

Interestingly, CA exPA patients tend to be much older than PA patients [11]. Since it is known that immune dysfunction increases with age (immunosenescence) and is closely related to the development of cancer, these data are consistent with a critical role of the immune system in the development of CA exPA.

In conclusion, adaptive immune surveillance may constrain the evolution of pleomorphic adenomas to malignancy. CA ex PA tumors have immunologically cold microenvironments compared with their *de novo* counterparts which may, in part, explain their more aggressive clinical behavior.

Further research will shed light on the specific immunological mechanisms and cellular populations that mediate this phenotype. As our patient cohort only comprised carcinomas and not adenomas, we can only speculate on the composition of the immune microenvironment in PA and how it evolves over time; ultimately, separate sampling of the PA and carcinoma compartments of a CA exPA tumor may be a more informative technique to dissecting genetic and immunologic factors mediating cancer evolution.

We also await further clinical data from ongoing clinical trials that may inform whether SGCs that are *de novo* carcinomas are more likely to respond to immunotherapeutic approaches, having more infiltrated immune microenvironments; or less likely, having undergone immune escape earlier in their development. As therapies evolve, these data also suggest there may be opportunities for immunotherapeutic interception of PA tumors—if malignant transformation could be prevented or predicted, the practice of immediate surgical resection for all benign PA tumors in the salivary gland may prove unnecessary in the future.
